# Genomic Dissection Reveals Polyphyletic Origins and Recombination-Driven Diversification of O/K Antigen Loci in Foodborne *Vibrio parahaemolyticus* O4:KUT Strains

**DOI:** 10.3390/microorganisms14091910

**Published:** 2026-08-28

**Authors:** Peng Zhang, Lei Ji, Wei Yan, Liping Chen, Fenfen Dong

**Affiliations:** Huzhou Center for Disease Control and Prevention, 999 Changxing Road, Huzhou 313000, China

**Keywords:** *Vibrio parahaemolyticus*, antigen, O4:KUT, genetic structure, food safety

## Abstract

*Vibrio parahaemolyticus* is a leading cause of seafood-associated gastroenteritis worldwide. Traditional serotyping based on 13 O and 71 K antigens fails to classify many isolates, designated as K-untypable (KUT), whose genetic basis and evolutionary dynamics remain unclear. In this study, we conducted whole-genome sequencing of 47 O4:KUT strains from 40 clinical and 7 retail aquatic products (snail, river shrimp, *Macrobrachium rosenbergii*, etc.) to decipher the genomic diversity and structural variation in their O/K antigen loci. Phylogenomic analysis revealed a polyphyletic population structure spanning multiple sequence types, with O/K antigen loci classified into 13 distinct structural types comprising 149 biosynthetic genes. These clusters exhibited mosaic architectures and varied functional profiles. Notably, pervasive phylogenetic incongruence and robust recombination signals identified horizontal gene transfer as the primary mechanism driving O/K antigen loci diversification. Our findings reveal the evolutionary mechanisms of the foodborne O4:KUT serotype prevalent in local seafood and clinical samples from Huzhou, China, and provide a preliminary gene-signature framework that may inform the future development of molecular serotyping assays.

## 1. Introduction

*Vibrio parahaemolyticus* is a Gram-negative, halophilic bacterium that thrives in warm marine and estuarine environments. Its strain classification is primarily based on somatic (O) and capsular (K) antigens, which vary depending on environmental conditions [1]. Lipopolysaccharide (LPS) is an essential component of the outer membrane of Gram-negative bacteria, composed of lipid A, core oligosaccharide, and O-specific polysaccharide (O antigen) [2,3]. These bacteria may also produce capsular polysaccharide (CPS, or K antigen). Furthermore, the capsule confers protection against physical and chemical stresses, significantly improving bacterial persistence and adaptability in harsh environments without compromising the efficiency of intercellular genetic material transfer [4].

One study suggested that the O-side chain gene cluster on chromosome I (VP0214-VP0238 in strain RIMD2210633, homologous to *gmhD-rjg* in *Vibrio cholerae* O139 MO45) determines K antigen, though experimental evidence was not provided [5]. The O-antigen-encoding gene clusters are located at loci VP0190-0213 in chromosome I in strain RIMD2210633 [5]. Subsequent research involving gene deletion experiments confirmed that VP0214-VP0238 specifically governs K antigen rather than O antigen [6].

Since its 1996 outbreak, the O3:K6 serotype has been the most prevalent, establishing *V. parahaemolyticus* as a major global foodborne pathogen [7]. However, other serotypes—such as O4:K8, O4:KUT (K-untypable), and O3:K8—emerged [1,8]. Notably, O4:KUT surpassed O3:K6 in detection frequency by 2013. It remained the predominant serotype until 2020 [9]. In that year, O10:K4 was first detected in Huzhou and later replaced O3:K6 as the new dominant strain [10]. Recently, O4:KUT has ranked second only to O10:K4 in causing local foodborne outbreaks [11], indicating a potential transmission chain between seafood and human infections.

Serotype O4:KUT dominates clinical isolates, yet the mechanisms governing its antigen variation are largely uncharacterized. To date, only two studies have characterized the K antigen of the O4:KUT serotype, assigning the designations O4:KUT2 and O4:KUT-recAin, respectively [9,12]. This study collected 47 O4:KUT strains from clinical and food samples for further analysis. We performed whole-genome sequencing of these 47 strains and systematically investigated the structural characteristics, functional profiles and evolutionary relationships of their O/K-antigen gene clusters. This work provides a genomic foundation for developing molecular serotyping tools.

## 2. Materials and Methods

### 2.1. Sample Collection and Serotyping

*V. parahaemolyticus* was isolated from clinical and food from 2020 to 2024 in several regions of Huzhou, China. The isolation, identification, and serotyping of *V. parahaemolyticus* were performed as previously described [13]. Species identification of *V. parahaemolyticus* isolates was carried out with the VITEK 2 Compact system (bioMérieux, Lyon, France). Serological typing was conducted via slide agglutination assays using commercial antisera targeting 11 O-antigens and 65 K-antigens supplied by Denka Seiken Ltd. (Tokyo, Japan), and all operations strictly followed the manufacturer’s official protocols. The isolation details were provided in Table S1. Food samples included snail, silver carp, hairtail, river shrimp and *Macrobrachium rosenbergii*, and the isolation of *V. parahaemolyticus* was performed in accordance with the national food safety standard of China (GB 4789.7-2013) [14].

### 2.2. Whole-Genome Sequencing

A total of 47 O4:KUT (including 7 foodborne isolates)—were selected for genome sequencing. The methodologies for nucleic acid extraction, library preparation, sequencing, and data processing have been described in our previous publication [15]. Whole-genome sequencing was conducted on the Illumina NovaSeq PE150 platform. Reads were quality-trimmed via FASTP and assembled using SPAdes v3.13.2. All isolates achieved sequencing depth over 100× and 90.9–97.5% coverage against reference RIMD 2210633 (Table S1). Assembly statistics, including N50 and contig numbers, are provided in Table S1. The sequence type (ST) was determined for each isolate using mlst (version 2.23.0) based on housekeeping genes.

### 2.3. Phylogenetic Analysis

A phylogenetic tree was reconstructed using single nucleotide polymorphisms (SNPs) identified across all isolates. Variants were called against the *V. parahaemolyticus* RIMD 2210633 reference genome (GCF_000196095.1) via the Snippy pipeline (https://github.com/tseemann/snippy, accessed on 7 June 2026). Core SNP alignment was generated with snippy-core v.4.6.0, after which recombinant regions were removed from the alignment using Gubbins v.3.0.0. Final SNP extraction was performed using SNP-sites. A maximum likelihood phylogenetic tree was constructed using IQ-TREE based on the SNP alignment file, with the GTR + F+ASC + R4 substitution model. The resulting tree was visualized using iTOL, with outer ring color-coding representing distinct STs. The resulting alignment file was imported into BioEdit to calculate pairwise SNP distances among all isolates. The pairwise SNP distance matrix is presented in Table S4.

### 2.4. Retrieval and Analysis of Antigen Sequences

Antigen sequences extraction: For the accurate retrieval of full-length antigen loci from the genomic data of all O4:KUT isolates, genome-wide homology searching was performed using BLAST 2.5.0+ to screen and anchor the conserved flanking genetic markers *VP189* and *VP238* within each de novo assembled genome. These two flanking genes were targeted owing to their high evolutionary conservation and consistent adjacency to the O- and K-antigen biosynthesis gene clusters, which enables precise boundary definition of the antigen-encoding genomic region. After unambiguous identification and positional confirmation of *VP189* and *VP238* in all isolate genomes, an in-house custom Python 3.7.6 script was utilized to perform automated sequence extraction and truncation. Following rigorous manual validation of sequence completeness, all retrieved sequences were uniformly defined as the integrated O-antigen and K-antigen locus sequences for subsequent bioinformatic analysis.

Sequence alignment and phylogenetic analysis: For comparative genomic and evolutionary analysis of the extracted O- and K-antigen locus sequences across all O4:KUT isolates, multiple sequence alignment was first performed using MAFFT to generate whole-locus sequence alignments. Following the generation of standardized multiple sequence alignments, phylogenetic reconstruction was conducted using IQ-TREE, a maximum likelihood-based algorithm optimized for high-resolution microbial genomic phylogeny. The best-fit nucleotide substitution model was automatically selected via the function of IQ-TREE to maximize phylogenetic accuracy. Branch support values were assessed by ultrafast bootstrap approximation with sufficient replicates to evaluate the statistical reliability of the tree topology. The alignment file was used as input to calculate pairwise sequence similarities among the antigen sequences. The resulting similarity matrix is presented in Table S3. Pairwise similarities among isolates within Groups 1, 2, 5, 7, 10, 11, 12, and 13 are highlighted in yellow.

Antigenic structure: The full-length extracted O- and K-antigen genomic sequences were subjected to systematic structural and functional annotation. Briefly, Prokka was utilized for automated prokaryotic genome annotation to precisely predict the locations of coding sequences, identify functional antigen-related genes, and characterize unannotated hypothetical genes within each antigen locus. The annotation outputs were generated in standard GenBank (GBK) format. For intuitive visualization and comparative analysis of inter-strain locus architecture, the annotated GBK files containing full gene positional and functional information were imported into genoPlotR.

Gene function classification: Functional classification of antigen genes was performed following a previously described standardized annotation framework [16]. All identified antigen-associated genes were systematically categorized into four functional groups based on their predicted biological roles and molecular functions. The classification groups included biosynthesis pathway genes responsible for antigen sugar backbone synthesis, processing and transportation genes that mediate antigen modification, processing and extracellular transport, glycosyltransferase genes functioning in sugar chain assembly and antigen structural diversification, as well as unclassified genes with ambiguous or undefined functional annotations that could not be assigned to any of the above categories.

### 2.5. Evolution Mechanism Analysis

The mechanistic investigation of the antigen was conducted following previously established methods [16]. To trace the origin of exogenous inserted sequences, whole-sequence homology searches were conducted against the NCBI GenBank database using BLASTN (https://blast.ncbi.nlm.nih.gov/Blast.cgi, accessed on 23 January 2026), with the E-value threshold strictly set to 0 for high-precision screening. Potential recombination signals within antigen-coding sequences were subsequently detected via the Recombination Detection Program (RDP), which integrates seven independent algorithms for comprehensive recombination prediction. To eliminate false-positive results and ensure reliability, a recombination event was considered authentic only if it was consistently identified by at least three algorithms with a statistical significance threshold of *p* < 1 × 10^−10^.

## 3. Results

### 3.1. Phylogenomic Diversity and Source-Related Clustering of O4:KUT Strains

**Figure 1:**
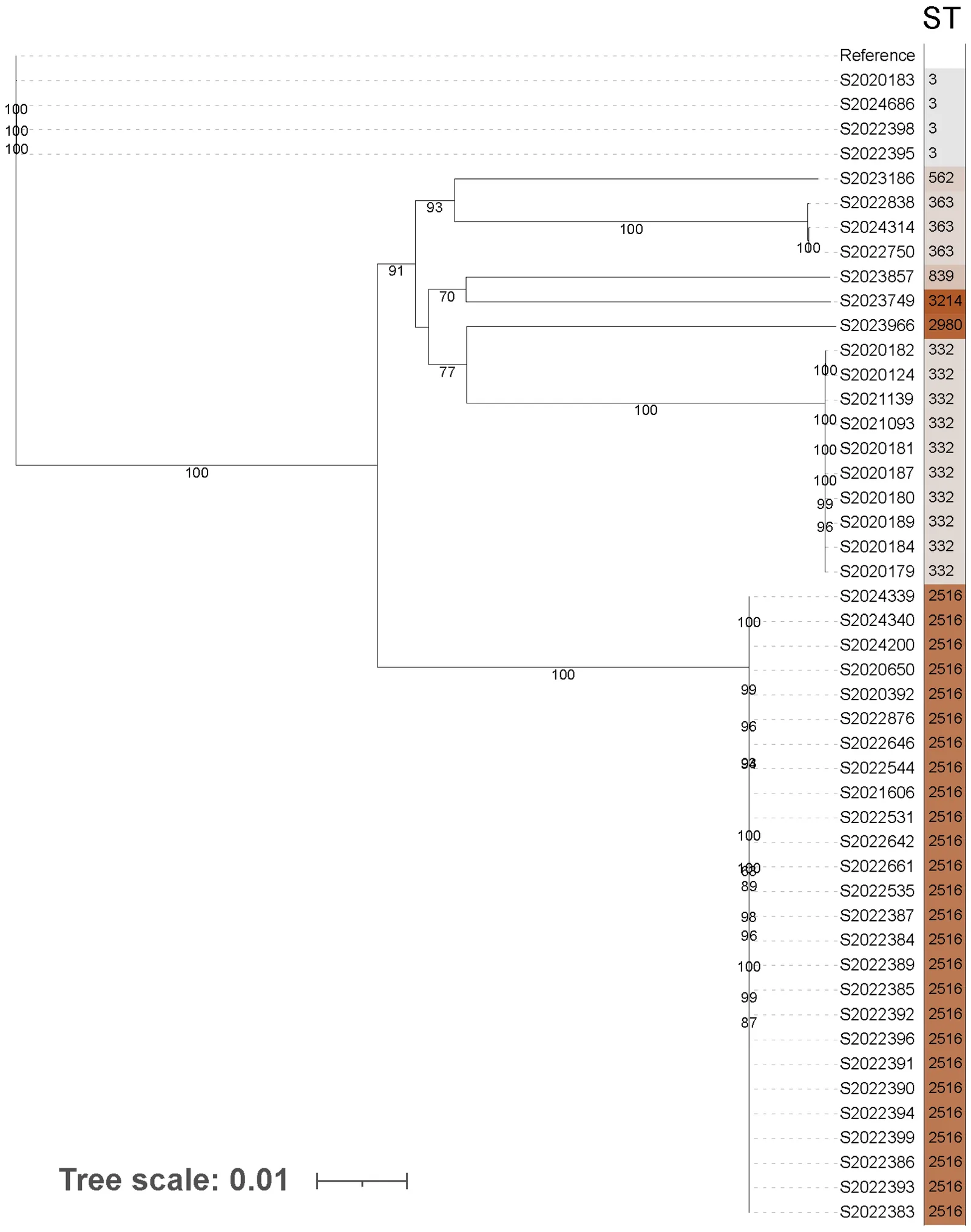
Phylogenetic tree of 47 *V. parahaemolyticus* O4:KUT isolates constructed based on whole-genome SNPs. The sequence types (STs) corresponding to each isolate are indicated on the right side of the tree.

To elucidate the genetic relatedness and evolutionary linkage between foodborne and clinical O4:KUT strains, as well as to evaluate their potential cross-host transmission along the food chain, a high-resolution core-genome single-nucleotide polymorphism (SNP)-based phylogenetic tree was constructed for a total of 47 O4:KUT isolates recovered from distinct clinical and food-associated sources. Genotype-based clustering demonstrated that clinical isolates were genetically concentrated and predominantly affiliated with three dominant sequence types, including ST3, ST332, and ST2516, suggesting that these ST lineages represent the major clinically prevalent genotypes of O4:KUT strains. In contrast, the 7 foodborne O4:KUT isolates exhibited substantially higher genetic polymorphism and scattered distribution, belonging to six phylogenetically distinct sequence types: ST363, ST562, ST3214, ST839, ST2980, and ST3. Overall phylogenetic clustering patterns were highly consistent with sequence type classification, with isolates of identical STs tightly grouped into independent clades. Compared with the clustered distribution of clinical strains, foodborne isolates displayed broader phylogenetic dispersion across the entire tree, reflecting a more diverse genetic background of food-circulating O4:KUT populations (Figure 1). To further investigate the potential transmission link between food sources and clinical cases, we analyzed pairwise SNP differences among all isolates. Notably, the minimum SNP distance between foodborne and clinical isolates within the ST3 and ST363 lineages was 10 (observed between the food isolate S2024686 and the clinical isolates S2022395 and S2022398). For the remaining ST3 and ST363 foodborne isolates, SNP distances to their clinical counterparts ranged from 47 to 197 (Table S4). These close genetic distances suggest the possibility of food-to-human transmission samples.

### 3.2. Antigen Gene Cluster Phylogeny Reveals Incongruence with Strain Evolution

**Figure 2:**
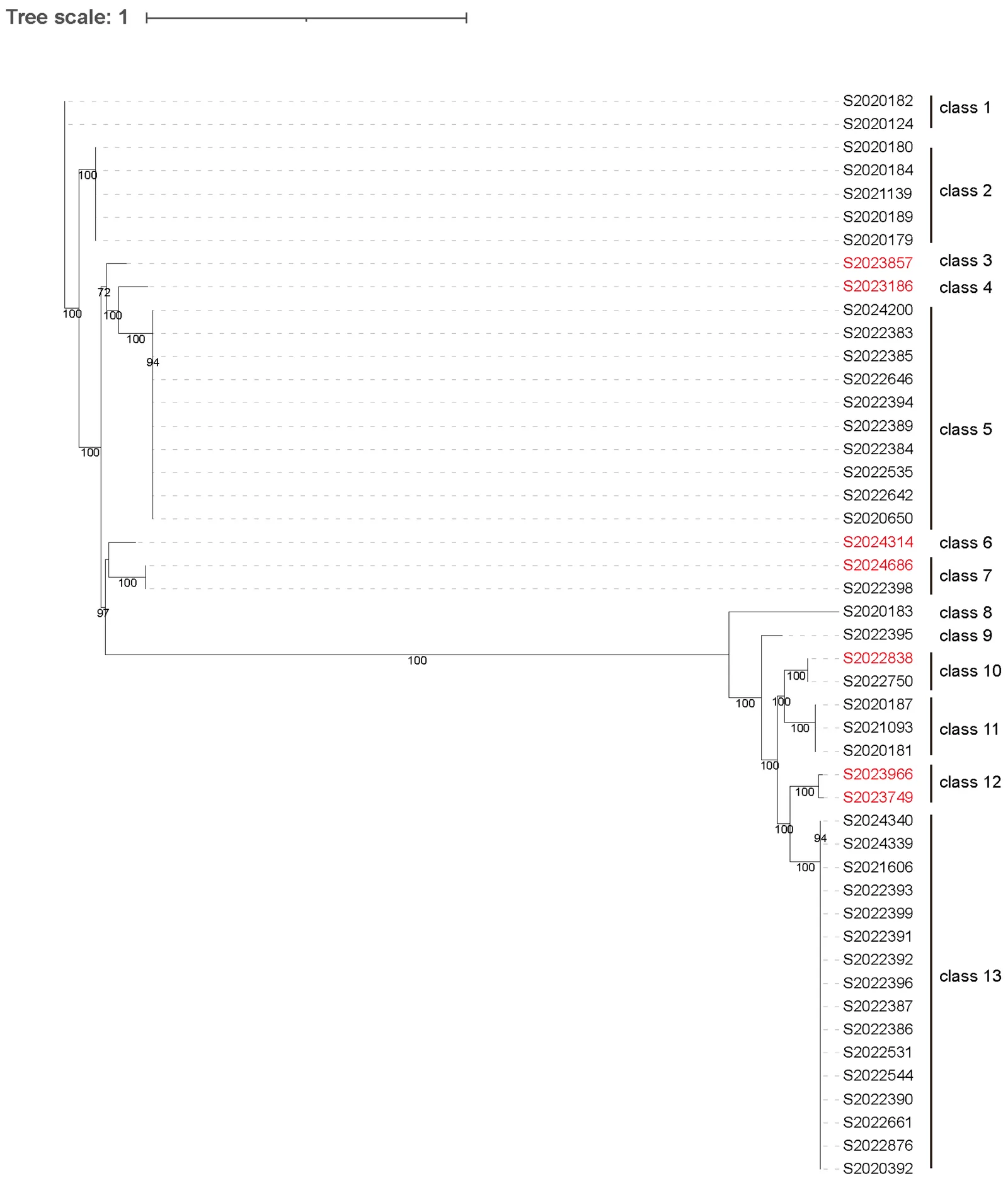
Phylogenetic tree of antigenic sequences from 47 isolates. Foodborne isolates are marked in red and clinical isolates in black.

To uncover the genetic determinants driving O/K antigen variation among O4:KUT strains derived from food and clinical sources, we systematically compared the phylogenetic relationships of O- and K-antigen gene clusters. Phylogenetic analysis of antigen loci clearly stratified these strains into 13 structurally divergent genotypes, revealing extensive genetic heterogeneity. Notably, the tree based on antigen gene clusters exhibited pronounced topological discordance with the core genome-based phylogeny, indicating inconsistent evolutionary trajectories between antigen loci and the overall genomic background. Representative incongruent cases were observed in pairwise strain comparisons: the foodborne isolate S2024686 harbored an identical antigen gene cluster structure to the clinically derived isolate S2022398, and likewise, the food isolate S2022838 shared highly consistent antigen locus organization with the clinical isolate S2022750 (Figure 2). Such remarkable phylogenetic incongruence between antigen profiles and whole-genome lineages strongly indicates that antigen-encoding gene cassettes can be independently acquired and exchanged via horizontal genetic recombination events among genetically distinct *V. parahaemolyticus* genomic backgrounds, which serves as a critical evolutionary mechanism facilitating the phenotypic variation in the O4:KUT serotype.

### 3.3. Genetic Architecture and Core-Pan Structure of Antigen Loci

**Figure 3:**
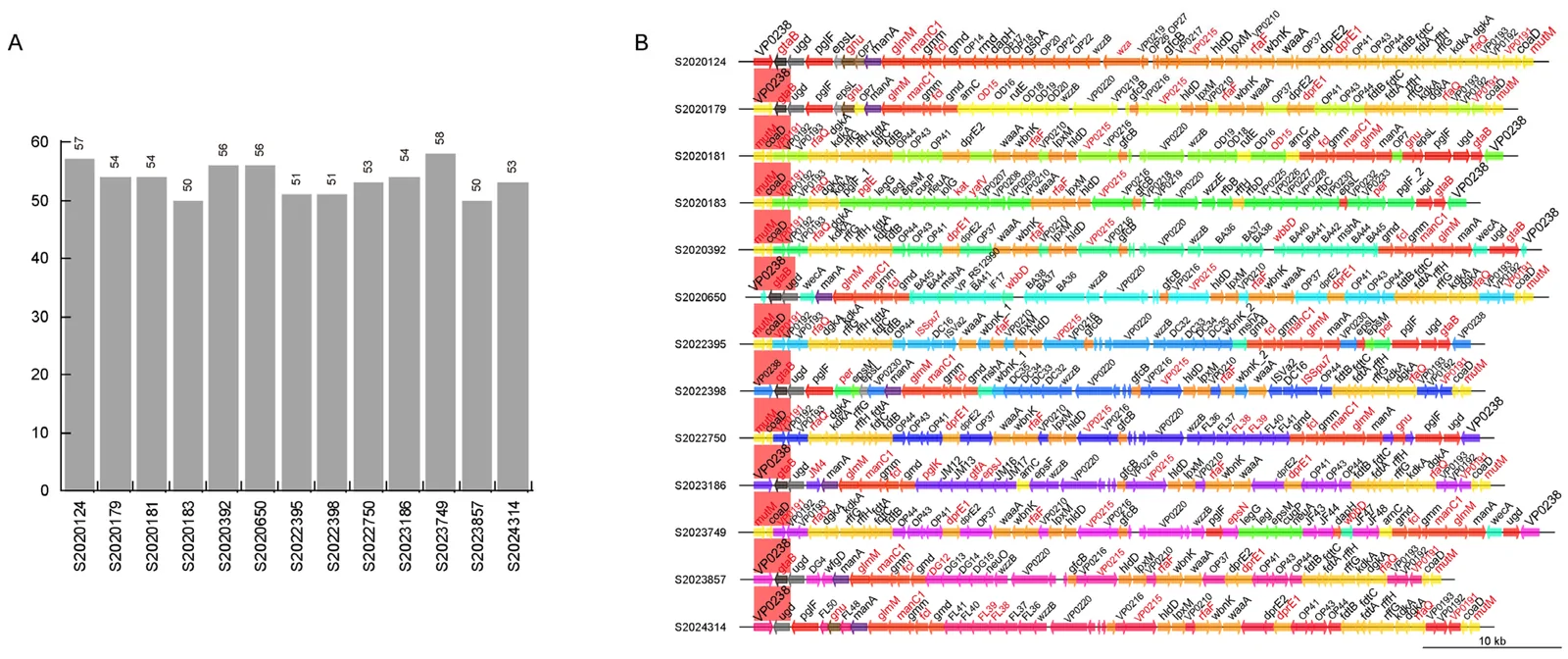
Genetic organization of the 13 O/K-antigen biosynthesis gene clusters. (**A**) Bar plot showing the number of open reading frames (ORFs) identified in each cluster. (**B**) Schematic representation of the 13 gene clusters. Each arrow represents a gene, with its direction indicating the transcriptional orientation. Homologous genes across clusters are color-coded. Exogenous insertion sequence (IS) elements are highlighted in red.

To validate the phylogenetic classification of the O/K antigen loci, we conducted pairwise nucleotide similarity analyses across all 47 sequences. Within each of the groups defined by the phylogenetic tree, the majority of pairwise comparisons exhibited 100% sequence identity, and the lowest intra-group similarity was 97.4% (Table S3), indicating that strains within the same group are essentially identical at the locus level. Comparative analysis of the 13 antigen locus types (each represented by a randomly selected strain) delineated their genetic organization. The flanking genes, notably *hldD* (*VP0214* homolog) and *coaD*, were highly conserved, framing the variable antigen-determining region. Genes with a distribution frequency > 90% across all isolates were defined as the core gene set. This included 19 ubiquitous (100%) genes (*hldD*, *ugd*, *mutM*, *coaD*, *rfaQ*, *dgkA*, *kdkA*, *waaA*, *rfaF*, *lpxM*, *wza*, *VP0238*, *VP0219*, *VP0217*, *VP0215*, *VP0210*, *VP0193*, *VP0192*, *VP0191*) and 15 near-ubiquitous (90–100%) genes (*gfcB*, *gmd*, *fcl*, *gmm*, *manC1*, *glmM*, *manA*, *rffG*, *rffH*, *fdtA*, *fdtC*, *fdtB*, *wzzB*, *OP44*, *VP0216*). Core genes were partitioned: those involved in flanking functions (e.g., *hldD*, *gfcB*, *rfaF*, *lpxM*) and those in central biosynthesis (e.g., *gmd*, *manC1*, *rfaQ*). The overall synteny of core genes was conserved, with a notable exception in isolate S2020183, where the *rffH* gene was translocated from the O- to the K-antigen region (Figure 3B). Putative origins of each gene were summarized in Table S2.

The number of open reading frames (ORFs) within the loci varied considerably (Figure 3A). The majority (*n* = 78) of K-antigen-specific genes were rare, present in fewer than three isolates, and primarily occupied the central variable region. Gene duplication events were observed; for example, *wbnK* and *pglF* existed in two copies in several serotypes, localized separately within O- or K-antigen regions (Table S2). Despite the conserved O4 serogroup, sequence variations, including deletions and replacements, were evident in the O-antigen regions, with isolate S2020183 showing the most extensive rearrangements (Figure 3).

### 3.4. Functional Characteristics of Antigen Sequences

**Figure 4:**
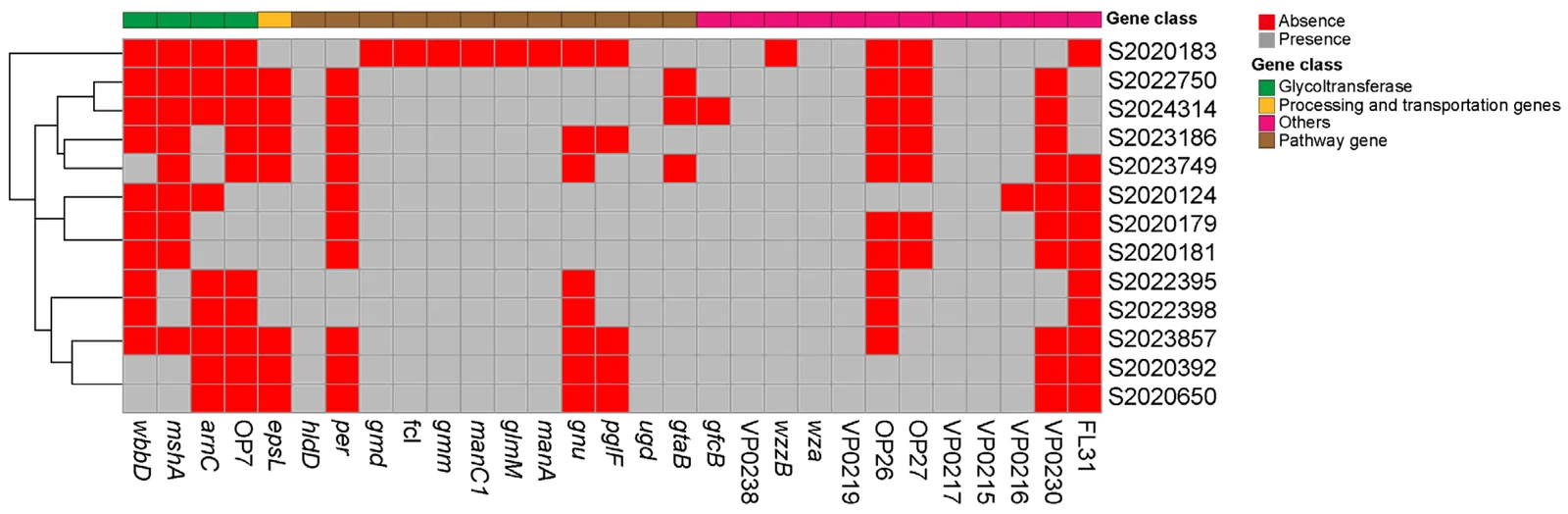
Prevalence of genes across isolates. The heatmap displays the presence (gray) or absence (red) of each K antigen gene that was detected in at least three isolates. Genes are grouped at the top by their functional category. Those genes existing in less than 3 isolations were not included; for all gene versions, please refer to Table S2.

Functional categorization of the annotated antigen genes revealed clear functional partitioning and uneven spatial distribution across the K-antigen locus. Gene functional classification demonstrated that core genomic regions were predominantly occupied by biosynthesis pathway-related genes. These core genes are highly conserved among different isolates and primarily encode key enzymes responsible for polysaccharide precursor synthesis, backbone polymerization, and fundamental antigen scaffold formation. Glycosyltransferase genes, which serve as the key genetic determinants of antigen structural variation and serological specificity, were predominantly enriched within the hypervariable central region of the antigen locus and exhibited relatively low overall distribution frequencies across the studied population. Besides major biosynthetic and specificity-determining genes, a small set of auxiliary processing and transportation genes was also identified within the antigen cluster; these genes are mainly localized in the intermediate transitional regions between the conserved core and hypervariable central segments of the K-antigen locus (Figure 3 and Figure 4, Table S2).

### 3.5. Recombination Hotspots Drive Antigen Locus Diversification

To further explore the key evolutionary mechanisms driving O/K-antigen structural diversification and phenotypic variation among foodborne and clinical O4:KUT strains, we systematically screened and characterized homologous recombination signals across the entire antigen locus and precisely mapped the genomic locations of recombination hotspots. Full-length aligned antigen locus sequences were subjected to robust recombination detection using the RDP5 software, v.5.80 suite. This preliminary screening yielded a total of 53 raw recombination signals across the dataset. To eliminate false-positive predictions and ensure analytical reliability, we applied strict stringent filtering criteria, requiring each validated recombination event to be simultaneously supported by no fewer than three independent detection algorithms with a statistical significance threshold of *p* < 1 × 10^−10^. After rigorous curation, a final set of 20 high-confidence, statistically robust recombination events was retained (Table 1). Further donor–recipient inference indicated that four representative strains, namely S2022395, S2022750, S2020181, and S2023749, frequently served as major genetic donors and recipients of recombinant fragments, highlighting their critical roles in mediating horizontal genetic exchange within O4:KUT populations. Spatial distribution analysis demonstrated that these confirmed recombination events were not randomly scattered across the antigen locus but were highly aggregated, forming four discrete recombination hotspots with distinct functional partitioning (Table 2). Each hotspot corresponded to independent antigen biosynthesis functional modules. Specifically, Hotspot A spanned genes primarily associated with cell wall remodeling and capsule regulatory functions, including *dapH*, *gspA*, and *wzzB*. Hotspot B covered core gene sets responsible for capsule assembly and LPS structural modification, consisting of *wza*, *gfcB*, *hldD*, *lpxM*, *rfaF*, *waaA*, and *wbnK*. Hotspot C was enriched in functional genes involved in enterobacterial common antigen and LPS biosynthesis, such as *rffH*, *rffG*, *dgkA*, *kdkA*, *and rfaQ*. Hotspot D was localized to the genomic region encoding enzymes for sugar precursor synthesis, represented by *manA* and *glmM*.

Two key patterns emerged: First, 90% (18/20) of events carried warnings of potential parent misidentification, indicative of recombination among highly similar sequences. Second, overlapping recombination intervals in hotspots A and B suggest either recurrent gene transfer at these loci or the inheritance of ancestral recombinant segments. These findings collectively establish homologous recombination, particularly within functionally coordinated gene blocks, as a primary mechanism generating antigenic diversity in O4:KUT populations.

## 4. Discussion

*Vibrio parahaemolyticus* is a major foodborne pathogen associated with seafood consumption, and serotype O4:KUT has become a predominant serotype in clinical diarrhea cases and retail aquatic products in Huzhou, China, in recent years [15]. The untypable K antigen of O4:KUT limits the efficiency of traditional serotyping in seafood safety surveillance and outbreak traceability in the aquatic product industry, making it urgent to elucidate the genetic basis and evolutionary mechanisms of this serotype for developing targeted control strategies. This study provides a high-resolution genomic analysis of O4:KUT strains from local retail aquatic products and clinical sources, revealing the polyphyletic origins and recombination-driven diversification of their O/K-antigen loci. These findings fill the research gap in genomic characteristics of O4:KUT strains in local aquatic product matrices and lay a genomic foundation for seafood safety surveillance of this serotype.

Building upon our previous identification of five STs among O4:KUT strains, the current phylogenomic analysis substantially expands the known diversity of this serotype to eight distinct STs: ST3, ST332, ST363, ST562, ST839, ST2516, ST2980, and ST3214. The polyphyletic distribution of these strains indicates multiple independent origins rather than clonal expansion from a single lineage, suggesting widespread contamination of O4:KUT strains in different aquatic product matrices and transmission routes in the local seafood production chain. Consistent with prior reports of genetic overlap between food and clinical reservoirs [17], the close phylogenetic affinity between selected food-derived and clinical isolates suggests a possible food-to-human transmission route, although this observation is based on a limited number of food isolates and requires confirmation through epidemiological data. The pathogenic ST3 lineage was exclusively identified in food, indicating that ST3 O4:KUT contamination in commercial seafood requires surveillance.

A key finding is the profound discordance between whole-genome phylogeny and antigen gene cluster phylogeny. While strains clustered by core genome similarity, their antigen loci were classified into 13 distinct structural types. This incongruence, exemplified by identical antigen profiles in genetically distant food and clinical strains (e.g., food isolate S2024686 and clinical isolate S2022398), strongly supports the horizontal acquisition of antigen cassettes. Such modular exchange, likely facilitated by extensive recombination within flanking genomic regions [18,19], enables rapid serotype switching without altering the core genomic backbone—a strategy that may enhance the adaptability of O4:KUT strains to different aquatic product matrices and food processing environments, as well as immune evasion in human hosts. This serotype switching ability also increases the difficulty of seafood safety surveillance, which further underscores the necessity of developing gene-based molecular serotyping assays instead of traditional serotyping methods. To directly compare our structural classification with previously reported subtypes, we retrieved the whole-genome sequences of O4:KUT2 and O4:KUT-recAin from public repositories and subjected them to the same antigen extraction and annotation. The results showed that O4:KUT2 shares an identical antigen locus structure with our S2020179, while O4:KUT-recAin matches our S2020181 (Table S2).

Structurally, the antigen loci follow a core-pan genome architecture. A conserved set of pathway genes, essential for synthesizing common polysaccharide precursors (e.g., UDP-glucose, UDP-GlcNAc, GDP-mannose), forms the stable scaffold for K-antigen biosynthesis in O4:KUT strains [20,21]. By comparison, the central hypervariable region harbors rare glycosyltransferases governing K-antigen specificity and diversity. Whereas the conserved core maintains basal growth and K-antigen synthesis in aquatic environments, variable glycosyltransferase modules facilitate strain adaptation to seafood-chain stresses such as disinfectants and high salinity, representing a vital food-adaptive trait of O4:KUT strains. The dynamic diversification of these loci is driven predominantly by homologous recombination, as evidenced by 20 high-confidence recombination events localized within four functional hotspots. Hotspot B, encompassing genes critical for capsule assembly and LPS modification (*wza*, *gfcB*, *lpxM*, etc.), appears particularly prone to transfer, suggesting that co-adapted gene complexes may be exchanged as functional units [22]. Recombination hotspots in the K-antigen locus (e.g., Hotspot B associated with capsule assembly) may enhance the adaptability of O4:KUT strains to food processing conditions (e.g., sanitizer exposure, high salt) and different seafood matrices (e.g., freshwater shrimp, snail), thereby increasing their persistence in the seafood production chain and the risk of human infection via seafood consumption. This targeted recombination facilitates coordinated adaptation of cell envelope properties, potentially conferring advantages under selective pressures such as host immunity and disinfectant treatment in aquatic product processing [23]. Acquisition of whole gene modules allows O4:KUT to remodel surface antigens rapidly for immune escape and environmental tolerance without disrupting envelope stability, a core survival strategy supporting its circulation along seafood chains [24].

We acknowledge that 90% of recombination events carry “potential parent misidentification” warnings. This affects directionality, not hotspot location, which remains robust. Accordingly, we interpret this genetic pattern as frequent, bi-directional gene exchange among closely related lineages. Future studies with a broader reference panel will help resolve true directionality. From a seafood safety perspective, the delineation of 13 genetically distinct O/K-antigen types in O4:KUT strains provides specific gene signatures for developing PCR-based serotyping tools. This proof-of-concept system demands systematic validation for practical use, including large-scale PCR verification across all 13 O4:KUT structural types and blind tests against standard serotyping methods. Central hypervariable glycosyltransferases are excellent primer markers owing to conserved sequences within types and high discrimination between types.

A major limitation of this study is that the phylogenetic and structural analyses of the antigen loci were performed on the concatenated O- and K-antigen sequences, rather than on independently extracted O- and K-antigen loci. Consequently, the 13 structural types and recombination events described here reflect the combined genetic variation in both the O- and K-antigen regions, and we cannot definitively determine the relative contribution of each locus to the observed diversity. Future studies will be necessary to resolve the independent evolutionary trajectories of the O- and K-antigen loci in O4:KUT strains. Other limitations of this study include the relatively small number of foodborne isolates (n = 7) and the geographically restricted sampling (Huzhou area). It is important to note that our conclusions regarding O/K-antigen diversity are based on predictions from genomic sequences. In addition, future research will focus on the development and validation of portable rapid detection kits based on the identified gene signatures, to realize on-site rapid detection of O4:KUT strains in the aquatic product industry.

## 5. Conclusions

In conclusion, this study deciphers the genomic diversity, O/K-antigen locus structure, and evolutionary mechanisms of the O4:KUT serotype prevalent in Huzhou. It confirms polyphyletic origins and recombination-driven diversification, with horizontal gene transfer as the core evolutionary force. This work fills the genomic gap for O4:KUT in local seafood, links traditional serotyping with genomic epidemiology, and provides a foundation for detection, traceability, and risk management.

## Figures and Tables

**Table 1:** High-confidence recombination events detected in 13 antigen sequences.

Event	Begin	End	Recombinant Sequence(s)	Major Parental Sequence(s)	Detection Methods						
					RDP	GENECONV	Bootscan	Maxchi	Chimaera	SiSscan	3Seq
1	75,556	83,576	^ S2023749	S2022750	2.86 × 10^−298^	5.42 × 10^−305^	1.72 × 10^−180^	8.58 × 10^−198^	8.58 × 10^−198^	1.17 × 10^−81^	9.1 × 10^−37^
2	46,808	52,510	^ S2020183	S2020179	1.53 × 10^−213^	NS	1.38 × 10^−145^	8.58 × 10^−198^	8.58 × 10^−198^	2.43 × 10^−35^	1.94 × 10^−6^
3	79,867	81,229 *	^ S2022395	S2022750	2.31 × 10^−218^	8.87 × 10^−157^	1.75 × 10^−203^	8.58 × 10^−198^	6.25 × 10^−51^	9.71 × 10^−56^	7.01 × 10^−5^
4	22,259	35,652	^ S2022750	S2022395	8.94 × 10^−189^	1.85 × 10^−215^	1.20 × 10^−225^	8.58 × 10^−198^	8.58 × 10^−198^	2.15 × 10^−73^	7.34 × 10^−28^
5	22,289	35,652	S2022395	S2020181	8.89 × 10^−177^	3.87 × 10^−210^	2.98 × 10^−177^	8.58 × 10^−198^	8.58 × 10^−198^	3.12 × 10^−71^	9.16 × 10^−32^
6	38,984	43,863	^ S2020179	S2020124	1.34 × 10^−120^	5.18 × 10^−200^	3.20 × 10^−124^	8.58 × 10^−198^	8.58 × 10^−198^	5.11 × 10^−39^	4.62 × 10^−135^
7	75,556	76,915	^ S2022395	Unknown (S2020392)	7.21 × 10^−161^	4.34 × 10^−178^	3.11 × 10^−155^	8.58 × 10^−198^	1.24 × 10^−36^	2.05 × 10^−56^	6.10 × 10^−21^
8	10,756	12,266	^ S2024314	Unknown (S2020124)	7.20 × 10^−107^	7.42 × 10^−95^	4.42 × 10^−108^	8.58 × 10^−198^	2.01 × 10^−29^	2.40 × 10^−53^	1.67 × 10^−79^
9	41,420	49,808	^ S2020392	Unknown (S2020181)	9.35 × 10^−95^	3.39 × 10^−117^	3.95 × 10^−47^	8.58 × 10^−198^	8.58 × 10^−198^	1.10 × 10^−38^	1.37 × 10^−11^
10	39,924	40,854	^ S2022395	Unknown (S2020392)	3.37 × 10^−67^	1.61 × 10^−75^	6.73 × 10^−57^	5.76 × 10^−22^	8.73 × 10^−18^	2.96 × 10^−35^	NS
11	10,777	12,259	^ S2022398	Unknown (S2020179)	1.84 × 10^−86^	2.10 × 10^−93^	NS	8.58 × 10^−198^	2.76 × 10^−28^	5.41 × 10^−55^	1.91 × 10^−13^
12	80,315	84,400	^ S2020181	Unknown (S2020392)	9.15 × 10^−72^	9.99 × 10^−72^	3.56 × 10^−84^	8.58 × 10^−198^	8.58 × 10^−198^	1.14 × 10^−72^	1.83 × 10^−3^
13	4128	9466	^ S2020181	S2022750	5.73 × 10^−49^	1.90 × 10^−61^	9.19 × 10^−62^	2.15 × 10^−22^	4.72 × 10^−17^	6.90 × 10^−22^	9.53 × 10^−14^
14	50,784	51,400	S2023749	S2020181	2.58 × 10^−40^	3.35 × 10^−54^	1.27 × 10^−45^	1.43 × 10^−17^	4.37 × 10^−18^	9.04 × 10^−20^	4.78 × 10^−11^
15	69,588	71,836	^ S2020181	S2020392	1.02 × 10^−40^	1.60 × 10^−40^	9.15 × 10^−51^	8.58 × 10^−198^	1.20 × 10^−13^	7.50 × 10^−25^	1.23 × 10^−10^
16	82,786	83,560 *	^ S2020124	S2023857	1.10 × 10^−15^	1.16 × 10^−10^	NS	3.42 × 10^−15^	NS	1.33 × 10^−28^	9.53 × 10^−14^
17	75,347	36,067 *	^ S2020181	S2022395	1.15 × 10^−12^	7.16 × 10^−11^	4.31 × 10^−13^	3.05 × 10^−12^	1.28 × 10^−10^	3.50 × 10^−8^	5.21 × 10^−4^
18	44,668	87,680	^ S2020181	Unknown (S2022395)	1.61 × 10^−10^	NS	NS	8.58 × 10^−198^	NS	2.78 × 10^−16^	3.42 × 10^−6^
19	49,103 *	52,371 *	^ S2020124	Unknown (S2022398)	9.97 × 10^−4^	2.39 × 10^−9^	9.92 × 10^−13^	2.10 × 10^−8^	1.30 × 10^−5^	5.07 × 10^−14^	NS
20	37,689	43,218	^ S2022398	S2023186	1.47 × 10^−5^	6.16 × 10^−14^	3.29 × 10^−12^	8.21 × 10^−6^	4.71 × 10^−3^	2.97 × 10^−10^	NS

* = The actual breakpoint position is undetermined (it was most likely either overprinted by a subsequent recombination event or off the edges of the analysed sequence fragments). ^ = The recombinant sequence may have been misidentified (one of the identified parents might be the recombinant) Major Parent = Parent contributing the larger fraction of sequence. Unknown = Only one parent and a recombinant need be in the alignment for a recombination event to be detectable. The sequence listed as unknown was used to infer the existence of a missing parental sequence. NS = No significant *p*-value was recorded for this recombination event using the particular method in question.

**Table 2:** Four recombination hotspot regions and their associated genes.

Hotspots	Regions	Covered Genes
A	22,000–36,000	*dapH*, *gspA*, *wzzB*, *OP17*, *OP18*, *OP20*, *OP21*, *OP22*
B	39,000–52,000	*wza*, *gfcB*, *hldD*, *lpxM*, *rfaF*, *waaA*, *wbnK*, *VP0219*, *OP26*, *OP27*, *VP0217*, *VP0215*, *VP0210*,
C	75,500–84,500	*rffH*, *rffG*, *dgkA*, *kdkA*, *rfaQ*, *VP0192*, *VP0193*
D	10,700–12,300	*manA*, *glmM*

## Data Availability

The sequence data have been deposited at NCBI under the BioProject PRJNA1393258.

## Supplementary Materials

The following supporting information can be downloaded at https://www.mdpi.com/article/10.3390/microorganisms14091910/s1. Table S1: Isolation information of the 47 *Vibrio parahaemolyticus* O4:KUT isolates; Table S2: Putative origins and associated information of antigen-coding genes. Table S3: Pairwise similarity matrix of the antigen sequences from 47 isolates. Table S4: Pairwise SNP distance matrix of the 47 isolates.
